# Effect of the Solvent on Propolis Phenolic Profile and its Antifungal, Antioxidant, and In Vitro Cytoprotective Activity in Human Erythrocytes Under Oxidative Stress

**DOI:** 10.3390/molecules25184266

**Published:** 2020-09-17

**Authors:** Magdalena Woźniak, Lucyna Mrówczyńska, Patrycja Kwaśniewska-Sip, Agnieszka Waśkiewicz, Piotr Nowak, Izabela Ratajczak

**Affiliations:** 1 Department of Chemistry, Faculty of Forestry and Wood Technology, Poznań University of Life Sciences, Wojska Polskiego 75, 60625 Poznań, Poland; magdalena.wozniak@up.poznan.pl (M.W.); agnieszka.waskiewicz@up.poznan.pl (A.W.); 2 Department of Cell Biology, Institute of Experimental Biology, Faculty of Biology, Adam Mickiewicz University in Poznań, Uniwersytetu Poznańskiego 6, 61614 Poznań, Poland; 3 Air Quality Investigation Department, Łukasiewicz Research Network–Wood Technology Institute, Winiarska 1, 60654 Poznań, Poland; p_kwasniewska@itd.poznan.pl; 4 Institute of Chemical Wood Technology, Faculty of Forestry and Wood Technology, Poznań University of Life Sciences, Wojska Polskiego 38/42, 60637 Poznań, Poland; 5 Institute of Economic Sciences, Wrocław University, Uniwersytecka 22/26, 50145 Wrocław, Poland; piotr.nowak2@uwr.edu.pl

**Keywords:** propolis, phenolic compounds, antifungal properties, antioxidant activity, human erythrocytes, oxidative hemolysis protection

## Abstract

Propolis is a natural bee product with various beneficial biological effects. The health-promoting properties of propolis depend on its chemical composition, particularly the presence of phenolic compounds. The aim of this study was to evaluate the relationship between extraction solvent (acetone 100%, ethanol 70% and 96%) and the antifungal, antioxidant, and cytoprotective activity of the extracts obtained from propolis. Concentrations of flavonoids and phenolic acids in the propolis extracts were determined using ultrahigh-performance liquid chromatography. The antioxidant potential of different extracts was assessed on the basis of 2,2-diphenyl-1-picrylhydrazyl (DPPH·) free-radical-scavenging activity, Fe^3+^-reducing power, and ferrous ion (Fe^2+^)-chelating activity assays. The ability of the extracts to protect human red blood cell membranes against free-radical-induced damage and their antifungal activity was also determined. The results showed that the concentration of flavonoids in the propolis extracts was dependent on the solvent used in the extraction process and pinocembrin, chrysin, galangin, and coumaric acid were the most abundant phenols. All extracts exhibited high antioxidant potential and significantly protected human erythrocytes against oxidative damage. On the other hand, the antifungal activity of the propolis extracts depended on the solvent used in extraction and the fungal strains tested. It needs to be stressed that, to the best of our knowledge, there is no study relating the effect of solvent used for extraction of Polish propolis to its phenolic profile, and its antifungal, antioxidant, and cytoprotective activity.

## 1. Introduction

Propolis is a natural product collected by honeybees from various plant sources growing around hives, including flowers, buds, or tree exudates [1,2,3]. Extracts of propolis have been used in folk medicine since early times for numerous purposes, especially as a remedy for various diseases [4,5,6]. Currently, propolis is being extensively investigated in various applications, especially in medicine, pharmacy, cosmetology, and the food industry [1,3]. It is used as a component of functional food, dietary supplements, preparations for cold symptoms, or dermatological preparations used in wound healing [6,7]. The wide spectrum of propolis applications results from its versatile biological and pharmaceutical activity. Propolis has been proven to exhibit, e.g., antifungal, antibacterial, anticancer, and antioxidant properties [6,7,8,9,10,11,12,13,14,15].

According to the literature data, the biological activity of propolis is mainly attributed to the presence of phenolic compounds, especially flavonoids, phenolic acids, and their esters [1,6,10]. For this reason, the phenolic fraction is the most extensively investigated among all constituents determined in propolis samples collected from various geographical regions [3,9,12,14]. Phenolic compounds regularly determined in poplar-type propolis samples include pinocembrin, galangin, chrysin, kaempferol, catechin, quercetin, rutin, pinostrobin, pinobanksin, naringenin, caffeic acid, coumaric acid, ferulic acid, caffeic acid phenethyl ester, or caffeic acid cinnamyl ester [11,14,15,16,17,18]. Chrysin, galangin, and pinocembrin are common flavonoids identified in propolis samples from various regions of Poland [4,8,9,19,20]. In addition, hesperetin, apigenin, kaempferol, naringenin, pinobanksin, quercetin, and phenolic acids, including caffeic, coumaric, ferulic, vanillic, gallic, and genistic acids, have also been identified in Polish propolis [4,8,9,19,20,21]. Fatty acids, ketones, enzymes, sterols, terpenes, minerals, and coumarins were also identified in propolis samples of various botanical origin [6,11,22,23,24,25,26,27,28,29]. The chemical composition and, therefore, the biological effect of propolis extracts depends on various factors, including the botanical origin of the raw material, the method of harvest, season of the year, or solvent used in the extraction process [2,14,28].

Over the past few years, the biological activities of Polish propolis were studied intensively, and its chemopreventive, antioxidant, antibacterial, antiproliferative, and antifungal activity was observed [4,5,8,9,30,31,32,33]. Moreover, propolis collected from an apiary located in the central–western part of Poland effectively protected human red blood cells (RBC) in vitro against 2,2′-azobis(2-methylpropionamidine) dihydrochloride (AAPH)-induced oxidative hemolysis [19,34]. To the best of our knowledge, the impact of the solvent used for the extraction of Polish propolis on its phenolic profile and the antifungal and antioxidant properties, especially on the protective effect of extracts against in vitro oxidative hemolysis of human erythrocytes, has not been reported to date. Therefore, the main purpose of this research was to evaluate the concentrations of phenolic compounds and the biological activity of propolis from Poland in relation to the solvent used for extraction of the raw material. The relationship between the propolis solvent and the antifungal, antioxidant, and cytoprotective activity of obtained extracts was also analyzed. The choice of solvent (acetone 100%, ethanol 70% and 96%) for propolis extraction was based on our previous studies, which analyzed the effect of the solvent used for extraction of propolis on its antimicrobial activity [35]. Moreover, this study, with the use of statistical analysis, attempted to identify the specific compounds from the phenolic fraction, the presence of which would be related to the biological activity of propolis extracts.

The identification of individual compounds responsible for the biological activity of propolis extracts would be a significant contribution to the standardization process of this material.

## 2. Results

### 2.1. The Concentration of Phenolic Compounds in Propolis Extracts

The extraction efficiency of propolis varied depending on the solvent used for the extraction process as follows: acetone—58.15%, 70% EtOH—59.10%, and 96% EtOH—66.70%. The concentrations of selected phenolic compounds, namely, flavonoids and phenolic acids, in the propolis extracts are presented in Table 1 and Table 2, respectively.

Flavonoid concentrations in the tested extracts varied depending on the solvent used in the extraction process. Statistical analysis of flavonoid concentrations indicated that only two of the eight detected compounds, namely, naringenin and pinobanksin, were found in all extracts at the same statistical level. Pinocembrin was the most abundant flavonoid in all the extracts and its concentration was the highest when 70% EtOH was used as the solvent (EEP70). Galangin and chrysin were also detected in all propolis extracts at high concentrations. In addition, EEP96 contained a relatively high concentration of kaempferol. The propolis extract obtained using acetone as a solvent (EPA) was characterized by the highest sum of flavonoids (95.25 mg/g of propolis extract) among the tested extracts. On the other hand, the sum of flavonoids in EEP70 was equal to 97.77% of that obtained for EPA and it was almost 20% higher than obtained for EEP96. The galangin concentration was the highest in EPA, whereas chrysin and pinocembrin concentrations were the highest in EEP70, and the highest concentration of kaempferol was detected in EEP96. The concentrations of rutin, epicatechin, catechin, genistein, pinostrobinm and myricetin were below the detection limits of ultra high-performance liquid chromatography (UPLC) with photodiode array detection (PDA) and triple-quadrupole mass spectrometry (TQD) in all propolis extracts.The highest concentration in all propolis extracts among phenolic acids was observed for coumaric acid, and its concentration was statistically solvent-dependent. The concentration of cinnamic acid was also high and at the same statistical level in all the extracts. Concentrations of five out of seven phenolic acids detected differed statistically depending on the solvent used in the extraction process. In EEP96, the sum of phenolic acids was only about 4% higher than that detected in the other two extracts. Chlorogenic and sinapic acids were detected in none of the tested propolis extracts.

### 2.2. Antioxidant Potential, Hemolytic Potency, and Cytprotective Activity of Propolis Extracts in Human Erythrocytes

The antioxidant potential of the propolis extracts was evaluated using standard cell-free antioxidant assays, namely, 2,2-diphenyl-1-picrylhydrazyl (DPPH·) free-radical-scavenging activity, Fe^3+^-reducing power, and ferrous ion (Fe^2+^)-chelating activity. The results obtained are presented in Table 3.

All the propolis extracts exhibited a high and statistically similar free-radical-scavenging activity, Fe^3+^-reducing power, and ferrous ion (Fe^2+^)-chelating activity. The values of the antiradical activity of the propolis extracts toward DPPH· ranged from 73.05 (EEP70) to 77.70% (EPA) of the activity determined for the standard antioxidant Trolox, and they were over 90% of the level for another standard, butylated hydroxyltoluene (BHT). All the propolis extracts were characterized by a statistically similar reducing potential equivalent to approximately 62% of the value for Trolox and over 75% of BHT activity. The extract obtained with 96% EtOH as a solvent (EEP96) was characterized by a lower ferrous-ion-chelating activity and differed statistically from the two other extracts. Moreover, all the extracts exhibited a capacity to chelate Fe^2+^ corresponding to 43% and to 55% of ethylenediaminetetraacetic acid (EDTA) activity, used in this study as the ferrous ions standard chelator.

As shown in Table 4, neither hemolytic activity nor significant biconcave RBC shape modification were observed for all the propolis extracts. The RBC shape was mostly discocytic (like that observed for the control cells incubated in phosphate-buffered saline only) after both short- (1 and 4 h) and long-term (24 h) incubation with the propolis extracts (results not shown).

To study the cytoprotective activity of the propolis extracts under oxidative stress conditions, in vitro human RBC-based assays were applied. As presented in Table 4, all propolis extracts at the concentration of 0.05 mg/mL were able to significantly protect human RBCs against oxidative hemolysis induced by peroxyl radicals generated from 2,2′-azobis-(2-methylpropionamidine) dihydrochloride (AAPH). However, the EPA and EEP96 efficiency was statistically similar and higher compared to EEP70 efficacy. Moreover, EPA and EEP96 effectiveness was about 2.5 times higher than that of the standard antioxidant BHT.

**Figure 1:**
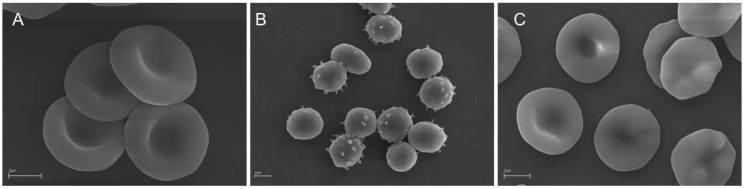
SEM micrographs showing human RBCs incubated (**A**) with phosphate-buffered saline (PBS buffer) for 5 h (control cells) and (**B**) with 60 mM AAPH for 4 h. (**C**) RBCs preincubated with the propolis extract (0.05 mg/mL) in 96% ethanol (EEP96) for 20 min and incubated with 60 mM AAPH for 4 h. Dominant RBC shape: (**A**) discocytes; (**B**) spheroechinocytes (spiculated spherocytes) and spherocytes (swollen, hemolytic RBCs); (**C**) discoechinocytes. The images show representative data after 1 h of independent experiments. Scale bars indicate 2 µm.

Simultaneously to the oxidative hemolysis assay, a microscopic analysis of the RBC shape was performed. As shown in Figure 1, following the preincubation of RBC with EEP96, the AAPH-induced morphological alteration of discoid (control) RBCs (Figure 1A) into echinospherocytes and spherocytes (Figure 1B) that undergo hemolysis was significantly reduced (Figure 1C).

One-way analysis of variance (ANOVA) was used to determine the effect of a single flavonoid concentration on the antioxidant potential of extracts. The corresponding *p*-values of the F test are summarized in Table 5. The average concentrations of naringenin and pinobanksin in all the propolis extracts were not statistically different in the THSD test (*p* < 0.05) and, therefore, these flavonoids were not included in this comparative analysis.

Statistical analysis showed that the flavonoid concentrations determined in the extracts did not affect the DPPH· free-radical-scavenging activity and the Fe^3+^-reducing power, while they showed an effect (except for galangin and quercetin concentrations) on the ferrous ion (Fe^2+^)-chelating activity of the propolis extracts. In addition, flavonoid concentrations had no influence on the cytoprotective potential of the extracts against oxidative hemolysis.

### 2.3. Antifungal Activity of Propolis Extracts

The effect of Polish propolis extracts on several molds was examined, and the results expressed as minimal inhibitory concentration (MIC) are presented in Table 6.

The tested mold strains exhibited various sensitivities to the propolis extracts. In this experiment, the values of the minimum inhibitory concentration (MIC) ranged from 0.5 mg/mL to 7.5 mg/mL. The intensity of the antifungal activity depended on the type of solvent and the species of fungi, with *Aureobasidium pullulans* being the most sensitive fungal strain toward all propolis extracts. Moreover, EPA and EPP70 showed higher activity (MIC = 0.5 mg/mL) against *A. pullulans* compared to the reference compound (MIC = 1.0 mg/mL). The antifungal effect of extracts was also noticeable in *Penicillium cyclopium*. The acetone propolis extract showed a higher inhibitory effect toward *Paecilomyces variotii* in comparison to the ethanolic extracts and a lower activity against *Penicillium funiculosum* than the other extracts. *Aspergillus niger* was the most resistant fungal strain to all the propolis extracts, with an MIC of 7.5 mg/mL.

In order to assess the effect of flavonoid concentrations in the propolis extracts on the antifungal activity, multivariate analysis of variance (MANOVA) was applied. The *p*-values of the Wilks test are given in Table 7. Naringenin and pinobanksin were not included in the MANOVA test, because their concentrations in all the propolis extracts were similar (according to THSD test (*p* < 0.05)).

The statistical analysis indicated that the concentrations of apigenin, chrysin, galangin, kaempferol, and pinocembrin in the propolis extracts significantly affected their antifungal activity.

## 3. Discussion

### 3.1. Concentrations of Phenolic Compounds in Propolis Extracts

Pinocembrin, galangin, and chrysin (Table 1), which were detected in all the tested propolis extracts at the highest concentrations, were previously reported in propolis collected from various regions of Poland, and they are regularly detected in propolis from around of the world [4,11,14,19,28,34,36,37,38,39]. Pinocembrin concentration in all the examined extracts (26.17–35.89 mg/g of propolis extract) was lower than in propolis extracts from Hungary (40.57–87.85 mg/g of propolis extract) and samples from the central–western region of Poland (41.55–51.55 mg/g of propolis extract) [19,37]. On the other hand, the concentration of pinocembrin in the tested propolis extracts was higher than in samples from Greece (0.4–14.0 mg/g of propolis extract), Italy (16.08–24.89 mg/g of propolis extract), and Ukraine (9.2 mg/g of propolis extract) [14,28,38]. Concentrations of galangin and chrysin in the propolis extracts (galangin: 15.08–23.91 mg/g of propolis extract and chrysin: 11.41–18.64 mg/g of propolis extract) were higher compared to samples from Greece (galangin: 0.02–2.53 mg/g of propolis extract and chrysin: 0.2–9.9 mg/g of propolis extract) and lower than in propolis collected from Bulgaria (galangin: 45.6 mg/g of propolis extract and chrysin: 66.3 mg/g of propolis extract) or Hungary (galangin: 44.2 mg/g of propolis extract and chrysin: 82.9 mg/g of propolis extract) [28,38]. The presence of phenolic acids (Table 2) detected in the examined propolis extracts was previously reported in propolis samples by other authors [4,9,11,31,36,38]. The concentration of coumaric acid determined in all the propolis extracts (7.90–9.56 mg/g of propolis extract) was higher than in propolis extracts from Hungary (0.13–1.02 mg/g of propolis extract), Greece (0.04–1.15 mg/g of propolis extract), and Bulgaria (3.5 mg/g of propolis extract) and similar to the contents in propolis from Italy (6.51–11.01 mg/g of propolis extract) and the Wielkopolska Province in Poland (9.17–10.04 mg/g of propolis extract) [14,19,28,37,38].

The qualitative and quantitative analyses of the phenolic profile in the propolis extracts indicated that the solvent used in the extraction process affected concentrations of flavonoids (Table 1) and, to a limited extent, those of phenolic acids (Table 2) in the propolis extracts. The effect of the solvent used to extract raw propolis on its chemical composition, especially the concentration of phenolic compounds, was also reported [2,40,41,42].

### 3.2. Antioxidant Properties and Cytoprotective Activity of Propolis Extracts in Human Erythrocytes

The examined propolis extracts showed a high antiradical potential, which was also characteristic of propolis extracts originating from different geographical regions, including samples from New Zealand, Brazil, Morocco, Hungary, Slovenia, India, or various parts of Poland [9,28,34,37,43,44,45,46]. The antiradical activity of all extracts (29.38–31.25%) confirmed in the DPPH· assay was higher than that observed for ethanolic extracts of propolis collected from the Wielkopolska Province of Poland (27.70–29.10%) [34]. The Fe^2+^-chelating activity of all the extracts (34.00–43.33%) was comparable to that of propolis extracts from central Poland (35.41–38.85%) [19,34]. Moreover, the propolis extracts exhibited a stronger chelating effect on ferrous ions than methanolic extracts of Portuguese propolis (4.33–29.68%) [47]. In turn, aqueous extracts of Portuguese propolis (41.11–82.35%) showed a higher chelating activity than all Polish propolis extracts [47]. The results presented in Table 3 indicate that the solvent used in the extraction process did not influence the DPPH· free-radical-scavenging activity or the Fe^3+^-reducing power of all the extracts. However, literature data showed that the solvent had an impact on the antioxidant properties of propolis extracts [2,14,40,41,42,46,48]. Miguel et al. [2] reported that the methanolic extract of Portuguese propolis showed higher free-radical activity than the aqueous extract. Papotti et al. [14] stated that the antioxidant activity of Italian propolis extract obtained with ethanol and acetone as solvents was higher than that of the chloroform extract. On the other hand, the ferrous ion (Fe^2+^)-chelating activity of our extracts was statistically dependent on the solvent (Table 3). These results are consistent with the literature data; specifically, the methanolic extracts of Portuguese propolis exhibited a lower chelating power than its aqueous extract [2]. Significantly lower ferrous-ion-chelating properties of EPP96 compared to those of the other extracts could be associated with a lower content of flavonoids (Table 1), which possess a strong iron-chelating activity [49]. According to the literature data, compounds such as caffeic acid, ferulic acid, galangin, quercetin, kaempferol, and cinnamyl caffeate exhibit high DPPH· free-radical-scavenging activity [28,46,50]. However, the statistical analysis showed no correlation between the concentration of individual flavonoids in the propolis extracts and their DPPH· free-radical-scavenging activity and Fe^3+^-reducing power (Table 5). The concentration of detected flavonoids, except for galangin and quercetin, had a statistically significant effect on the ferrous ion (Fe^2+^)-chelating activity of the propolis extracts. Therefore, taken together, it can be assumed that the beneficial activity of extracts is a result of a specific cooperation of different phenolic compounds.

Human RBCs are used as a cell model for screening of the antioxidant activity of bioactive compounds [19,34,51,52]. The evaluation of hemolytic activity of potential blood-contacting compounds is the most important criterion for their future in vivo applicability [53]. In this study, the hemocompatibility of the propolis extracts, namely, hemolytic activity and their effects on RBC shape, were analyzed with respect to their concentration (up to 0.1 mg/mL) and incubation time with cells (1, 4, and 24 h). As shown in Table 4, the propolis extracts did not exhibit RBC membrane-perturbing activity at 0.1 mg/mL; therefore, they were used for further studies as safe hemocompatible agents.

As indicated by data presented in Table 4, all the propolis extracts inhibited AAPH-induced hemolysis. However, EEP70 was statistically weaker as a cytoprotective agent under oxidative stress conditions. The statistically lower concentration of kaempferol in EEP70 was the only difference found. Many beneficial biological effects of kaempferol have been reported, including its antioxidant activity [54]. Therefore, we can speculate that the concentration of kaempferol can influence the activity of EEP70 as an antioxidant (Table 3) and a cytoprotective (Table 4) agent. However, according to the statistical results presented in Table 5, flavonoid concentrations had no effect on the cytoprotective potential of the extracts against oxidative hemolysis.

To estimate the protective efficiency of the propolis extracts against the discoid RBC shape alternation, simultaneously to the AAPH-induced hemolysis measurements, a scanning electron microscope analysis of the RBC shape was performed. As shown in Figure 1, SEM images present significant differences in the RBC morphology incubated with AAPH alone (Figure 1B) and preincubated with a propolis extract (EEP96) before incubation with AAPH (Figure 1C). The high ability of the propolis extracts to inhibit both AAPH-induced hemolysis and RBC shape transformation can be explained by their high antioxidant potency confirmed in cell-free assays (Table 3). However, it is known that bioactive compounds can be incorporated into RBC lipid bilayer without the echinocyte–stomatocyte transformation [55]. Therefore, the high efficiency of propolis extracts in the RBC protection against oxidative damage can be explained by (i) their antioxidant activity in the RBC solution (outside cells), and (ii) their presence in the cell membrane lipid bilayer (in the cell environment). Moreover, it should be stressed that the observed high cytoprotective activity is not only dependent on the chemical composition of extracts, but also on the specific interaction of extract components with the RBC membrane. Taken together, the interactions of flavonoids and phenolic acids with the RBC membrane are crucial in the cellular defense against oxidative stress conditions observed for all propolis extracts.

### 3.3. Antifungal Activity of Propolis Extracts

The values of the minimal inhibitory concentration (MIC) of fungal strains (Table 6) indicated that all the propolis extracts exhibited antifungal activity depending on the species of fungi. The literature confirms the antifungal action of propolis from various geographical regions against pathogenic fungi in humans, as well as fungi responsible for plant diseases [4,5,7,31,36,56,57,58]. The propolis extracts were effective against many fungal stains, such as *Aspergillus niger*, *Penicillium chrysogenum*, *Candida albicans*, *C. glabrata*, *Rhodotorula mucilaginosa*, *Fusarium solani*, *Botrytis cinera*, *P. notatum*, and *Saccharmyces cerevisiae* [4,5,7,31,57]. The highest MIC value (7.5 mg/mL) of all the tested extracts was observed for *A. niger*, which was comparable to the MIC values previously reported for propolis extracts from Poland [31,35]. The results of the antifungal activity showed that the solvent used for the extraction of propolis affected the antifungal activity, which is consistent with the literature data [5,45,48]. Garedew et al. [5] reported that the ethanolic extract of propolis was more active against *A. niger*, *Trichoderma viride*, and *Penicillium chrysogenum* than the aqueous extract. Mavri et al. [48] showed that the propolis extract obtained using 70% EtOH for extraction exhibited higher activity against molds and lower activity against yeasts in comparison to the extract when 96% EtOH was used as a solvent of propolis. Pinocembrin, chrysin, galangin, and pinostrobin are often reported in the literature as the components of propolis responsible for its antifungal activity [10,45,59]. According to the statistical analysis (Table 7), the concentrations of apigenin, chrysin, galangin, kaempferol, and pinocembrin in the propolis extracts significantly affected their fungistatic activity (Table 6), which is in agreement with literature data.

## 4. Materials and Methods

### 4.1. Chemicals and Reagents

Acetone and ethanol used to prepare propolis extracts were purchased from Avantor Performance Materials (Gliwice, Poland). All phenol standards (apigenin, chrysin, quercetin, galangin, myricetin, kaempferol, rutin, genistein, naringenin, epicatechin, pinocembrin, pinobanksin, pinostrobin, catechin, caffeic acid, ferulic acid, coumaric acid, sinapinic acid, cinnamic acid, vanillic acid, syringic acid, chlorogenic acid, and *p*-hydroxybenzoic acid) and chemicals (methanol, acetonitrile, and formic acid) used in UPLC analysis were purchased from Sigma Aldrich (Steinheim, Germany). Chemicals for the determination of antioxidant activity and cytotoxicity of the propolis extracts in human erythrocytes (dimethyl sulfoxide (DMSO), potassium ferricyanide, trichloroacetic acid, iron(III) chloride, iron(II) chloride, ferrozine, glucose, paraformaldehyde, glutaraldehyde) were purchased from Avantor Performance Materials (Gliwice, Poland). Butylated hydroxyltoluene (BHT), Trolox, 2,2-diphenyl-1-picrylhydrazyl (DPPH), 2,2′-azobis-(2-methylpropionamidine) dihydrochloride (AAPH), poly-l-lysine, ethylenediaminetetraacetic acid (EDTA), phosphate-buffered saline (PBS buffer: 137 mM NaCl, 2.7 mM KCl, 10 mM NaH_2_PO_4_, 1.76 mM KH_2_PO_4_)), and potato dextrose agar (PDA) were purchased form Sigma Aldrich (Steinheim, Germany).

### 4.2. Propolis and Preparation of Extracts

Raw propolis was purchased from a beekeeping company (Prokit–Miłosz Górecki, Halinów, Poland) and was originally collected from May to September 2014 in an apiary located in the Warmia-Masuria Province in northeastern Poland. Ground propolis samples (15 g) were extracted with 150 mL of acetone and ethanol at two different concentrations, i.e., 70% and 96%. The extraction was carried out under shaking (Biosan, Riga, Latvia) for 5 days at ambient temperature. The propolis extracts were then filtered through a Watman no. 4 filter paper (Sigma Aldrich, Steinheim, Germany) and solvents were evaporated using a rotary evaporator (Buchi Labortechnik AG, Flawil, Switzerland). Then, the extraction yield of propolis was calculated according to the following equation:

(1) $$Extraction\;yield\;\left(\%\right)=\frac{W_{dry\;extract\;}}{W_{raw\;propolis}}\cdot 100\%,$$

where *W_dry extract_* is the weight of the dry extract after solvent evaporation (g), and *W_raw propolis_* is the weight of raw propolis (g).

The obtained residues were next used in chemical and biological analyses.

### 4.3. Analysis of Phenolic Comopounds in Propolis Extracts

The residues of propolis extracts obtained in Section 4.2 were dissolved in methanol and used to determine the concentration of phenolic compounds using the Aquity UPLC chromatograph (Waters, Manchester, MA, USA) equipped with a photodiode detector (PDA eλ Detector) (Waters, Manchester, MA, USA) and coupled to an electrospray ionization triple-quadrupole mass spectrometer (TQD) (Waters, Manchester, MA, USA). All extracts of propolis were filtered through a 0.20 µm syringe filter (Chromafil, Macherey-Nagel, Duren, Germany) before analyses. The compounds were separated at 25 °C on a Waters ACQUITY UPLC HSS T3 column (150 × 2.1 mm/ID, with 1.8 µm particle size) (Waters, Manchester, MA, USA). Gradient elution was applied using water containing 0.1% HCOOH (A) and acetonitrile containing 0.1% HCOOH (B) with the flow rate of 300 µL/min. The solvent gradient was modified as follows: 0–5 min 25% B, 5–20 min 40% B, 20–30 min 60% B, 30–35 min 90% B, and 35–40 min 100% B, followed by a return to the initial conditions. The collision-induced decomposition was run using argon as the collision gas, with a collision energy of 25–40 eV. Multiple reaction monitoring (MRM) was used for quantitative analysis of compounds, which were identified by comparing the retention times and *m*/*z* values obtained by MS and MS^2^ with the mass spectra of the corresponding standards tested under the same conditions. The mass-to-charge ratios (*m*/*z*) of the parent and daughter ions, as well as the mode of ionization and the best collision energy, are presented in Table S1 (Supplementary Materials). All samples were injected in triplicate.

### 4.4. Antioxidant Activity of Propolis Extracts

The antioxidant activity of the propolis extracts was determined on the basis of three cell-free assays: (i) the DPPH· free-radical-scavenging activity, (ii) Fe^3+^-reducing power, and (iii) ferrous ion (Fe^2+^)-chelating activity, according to the methods described in our previous work [19]. In this study, the propolis extracts were used at a concentration of 0.1 mg/mL in PBS buffer. The stock solution of propolis (10 mg/mL) was obtained by dissolving the propolis residue in DMSO.

#### 4.4.1. DPPH· Free-Radical-Scavenging Activity

The solution of (0.1 mM) of DPPH· in ethanol (0.2 mL) was added to 0.2 mL of propolis extracts and vortexed (Bio Vortex V1, Biosan, Riga, Latvia). The reference antioxidants Trolox and BHT were used to determine the DPPH· free-radical-scavenging activity. The samples of reference compounds and propolis extracts were incubated in the dark for 30 min at room temperature. After this time, the absorbance (Abs) was measured at 517 nm, using an EPOLL 2000 ECO spectrophotometer (PZ EMCO, Warszawa, Poland). The percentage DPPH· scavenging effect was calculated using the following equation:

(2) $$DPPH\cdot scavenging\;activity\;\left(\%\right)=\frac{Abs_{0\;}-\;Abs_{1\;}\;}{Abs_{0}}\cdot 100\%,$$

where *Abs*_0_ is the absorbance of the control samples, and *Abs*_1_ is the absorbance of the tested samples. Every sample tube was prepared in triplicate, and every experiment was repeated three times. The results (*n* = 9) are presented as the mean value ± standard deviation (±SD).

#### 4.4.2. Fe^3+^-Reducing Power

The propolis extracts (0.06 mL) were mixed with 0.1 mL of 0.2 M PBS and 0.1 mL of 1% potassium ferricyanide. Trolox and BHT were used as reference compounds. The samples were vortexed (Bio Vortex V1, Biosan, Riga, Latvia) and incubated for 20 min at 50 °C. After incubation, 10% trichloroacetic acid (0.1 mL) and 0.6 M chloride irons (III) (0.04 mL) were added to each sample. Finally, the absorbance (Abs) was measured at 700 nm in an EPOLL ECO spectrophotometer (PZ EMCO, Warszawa, Poland). Every sample tube was prepared in triplicate, and every experiment was repeated three times. The results (*n* = 9) are presented as the mean value ± standard deviation (±SD).

#### 4.4.3. Ferrous Ion (Fe^2+^)-Chelating Activity

The propolis extracts (0.2 mL) were mixed with a solution of 0.6 mM chloride irons (II) (0.05 mL). EDTA was used as the standard metal chelator. The reaction was started by the addition of 5 mM ferrozine (0.05 mL) in ethanol, and the mixture was immediately vigorously shaken (Bio Vortex V1, Biosan, Riga, Latvia). The samples were stored for 10 min at room temperature and, after this time, the absorbance (Abs) of the samples was measured at 562 nm using an EPOLL ECO spectrophotometer (PZ EMCO, Warszawa, Poland). Every sample tube was prepared in triplicate, and every experiment was repeated three times. The percentage of inhibition of ferrozine–Fe^2+^ complex formation was calculated according to the following equation:

(3) $$Fe^{2+}chelating\;effect\;\left(\%\right)=\left[1-\frac{Abs_{1\;}\;}{Abs_{0}}\right]\cdot 100\%$$

where *Abs*_0_ is the absorbance of the control samples, and *Abs*_1_ is the absorbance of the tested samples. Every sample tube was prepared in triplicate, and every experiment was repeated three times. The results (*n* = 9) are presented as the mean value ± standard deviation (±SD).

### 4.5. In Vitro Effects of Propolis Extracts on Human Red Blood Cells (RBCs)

The hemolytic activity of the propolis extracts and their effects on the RBC morphology were studied on the propolis extracts at a concentration of 100 µg/mL. The analyses were carried out on fresh human erythrocyte concentrates purchased from the Blood Bank in Poznań, Poland, according to the methods described by Woźniak et al. [19]. The cytotoxicity of propolis extracts was estimated as the percentage (%) of hemolysis, and unfixed RBC shape transformation was examined under a Zeiss LSM 510 microscope Axiovert Zoom (ZEISS, Oberkochen, Germany). The cytoprotective potential (%) of propolis extracts against oxidative hemolysis induced by free radicals generated by thermolysis of 2,2′-azobis-(2-methylpropionamidine) dihydrochloride (AAPH) was estimated. Each sample tube was made in triplicate, and five independent experiments with RBCs from four different donors were performed using every single propolis extract. The results (*n* = 15) are presented as the mean value ± standard deviation (±SD).

### 4.6. Erythrocyte Shape Evaluation Using a Scanning Electron Microscope (SEM)

The RBCs treated as above were fixed in 5% paraformaldehyde (PFA) and 0.01% glutaraldehyde (GA). Following washing three times with PBS buffer (by gentle exchanging the supernatant with PBS), cells were fixed in 1% GA for 60 min at room temperature (RT). Following washing, RBCs were post-fixed with 1% OsO_4_ for 30 min at RT. Following the next washing, the RBCs were dehydrated in a series of ethanol solutions (50%, 60%, 70%, 80%, 90%, 95%, and 100%) in RT, critical-point-dried, and gold-sputtered. A large number of RBCs were examined using an EVO 40 (ZEISS, Oberkochen, Germany) scanning electron microscope.

### 4.7. Antifungal Properties of Propolis Extracts

The following fungal strains were used: *Aspergillus niger* van Tiegen ATCC 6275, *Aspergillus versicolor* ATCC 11730, *Paecilomyces variotii* ATCC 18502, *Penicillium funiculosum* ATCC 11797, *Aureobasidium pullulans* ATCC 9348, *Penicillium cyclopium* Westling, and *Trichoderma virens*. The species were provided by the BAM Federal Institute for Materials Research and Testing collection or by the Institute of Chemical Wood Technology (Poznań University of Life Sciences). The bioassay investigating the propolis extracts was prepared in a 96-microdilution tray using the broth microdilution method. The MIC (minimal inhibitory concentration) value was defined as the lowest concentration of the antifungal agent which resulted optically clear. The 96-well plates were prepared by dispensing 100 μL of double-concentrated potato dextrose agar culture medium into the each well. Then, 100 μL from their serial dilutions with different amounts of the propolis extracts (0.01–10 mg/mL) were transferred into the subsequent wells. To each well, 10 µL of freshly made fungal spore suspensions in concentrations of colony-forming units (CFU) from 10^−5^ to 10^−6^ CFU/mL were added using a microtiter. Those fungal spore suspensions were obtained from two-week agar slants. The results for each extract were compared to the control wells with a commercial fungicide 3-iodo-2-propynylbutylcarbamate (IPBC) as Preventol^®^ MP100 (Sigma Aldrich, Steinheim, Germany). The last well contained 200 μL of PDA with the fungal suspension to confirm the cell viability (viability control). Next, the plates were mixed on a plate shaker (300 rpm) for 30 s and incubated aerobically for 3–5 days in a moist chamber with relative humidity (RH) above 95% at 28 ± 1 °C in the dark. For reproducibility and accuracy evaluation of the microtiter plate method, the compounds were tested in triplicate.

### 4.8. Statistical Analysis

The results were analyzed using multivariate analysis of variance (MANOVA) applying the Wilks test and one-way analysis of variance (ANOVA) with Tukey’s honestly significant difference (THSD) test. All of the statistical analyses were performed using TIBCO Software Inc. Statistica version 13 (Palo Alto, CA, USA).

## 5. Conclusions

The qualitative and quantitative analysis of the phenol profile in the extracts of Polish propolis indicated that the solvent used in the extraction affected the concentration of flavonoids and, to a limited extent, the concentrations of phenolic acids. In all the propolis extracts, the most abundant phenolic compounds were pinocembrin, chrysin, galangin, and coumaric acid. The results of antioxidant assays indicated that all the extracts exhibited high DPPH· free-radical-scavenging activity, Fe^3+^-reducing power, and Fe^2+^-chelating activity. Moreover, the cytoprotective activity against free-radical-induced human erythrocyte hemolysis was observed for all the propolis extracts. Statistical data showed no correlation between the content of individual flavonoids in the propolis extracts and their DPPH· free-radical-scavenging activity and Fe^3+^-reducing power. Concentrations of flavonoids, except for galangin and quercetin, statistically affected the ferrous ion (Fe^2+^)-chelating activity of the extracts. All the propolis extracts showed antifungal properties, which were dependent on the type of solvent used and the fungal strains. According to the statistical results, the concentrations of apigenin, chrysin, galangin, and pinocembrin significantly affected the fungistatic activity of these propolis extracts.

In summary, this is the first study presenting the dependence between the solvent used in Polish propolis extraction and the extract composition, as well as its biological properties, including in vitro effects on human erythrocyte shape and membrane permeability under both physiological and oxidative-stress conditions. On the basis of our results, it can be stated that the solvent-dependent Polish propolis extracts possess antioxidant and antifungal activities that can be attributed to presence of phenolic compounds. The statistical analysis showed that chrysin, galangin, pinocembrin, apigenin, and kaempferol are phenolic compounds that significantly affect the antifungal activity of Polish propolis extracts in a dose-dependent manner. It can be concluded that the identification of compounds responsible for the biological activity of Polish propolis will contribute to the development of procedures for its standardization, thus ensuring high quality of this product. Moreover, it is very important to search for new bioactive substances in different solvent-extracted propolis extracts, especially in the context of increasing antibiotic resistance of pathogenic microorganisms.

## Figures and Tables

**Table 1:** Flavonoid concentrations in propolis extracts.

Flavonoids	Concentration (mg/g of Propolis Extract)		
	EPA (Acetone)	EEP70 (70% EtOH)	EEP96 (96% EtOH)
Apigenin	9.57 ^a^ ± 0.38	7.96 ^a^ ± 0.47	6.13 ^b^ ± 0.68
Chrysin	14.62 ^b^ ± 0.55	18.64 ^a^ ± 0.71	11.41 ^c^ ± 0.73
Galangin	23.91 ^a^ ± 0.71	16.18 ^b^ ± 0.42	15.08 ^b^ ± 0.27
Kaempferol	10.63 ^b^ ± 0.39	8.20 ^c^ ± 0.43	12.15 ^a^ ± 0.40
Naringenin	0.80 ^a^ ± 0.20	0.86 ^a^ ± 0.23	0.33 ^a^ ± 0.06
Pinobanksin	3.62 ^a^ ± 0.30	3.10 ^a^ ± 0.24	3.56 ^a^ ± 0.25
Pinocembrin	30.68 ^b^ ± 0.24	35.89 ^a^ ± 0.95	26.17 ^c^ ± 0.61
Quercetin	1.42 ^ab^ ± 0.29	2.30 ^a^ ± 0.43	1.30 ^b^ ± 0.18
Sum of flavonoids	95.25	93.13	76.13

Values in the same row followed by the same letter are not significantly different on the basis of Tukey’s honestly significant difference (THSD) test (*p* < 0.05).

**Table 2:** Concentrations of phenolic acids in propolis extracts.

Phenolic Acids	Concentration (mg/g of Propolis Extract)		
	EPA (Acetone)	EEP70 (70% EtOH)	EEP96 (96% EtOH)
Caffeic acid	2.23 ^a^ ± 0.31	2.54 ^a^ ± 0.37	2.15 ^a^ ± 0.18
Coumaric acid	9.19 ^a,b^ ± 0.55	7.90 ^b^ ± 0.57	9.56 ^a^ ± 0.32
Ferulic acid	1.63 ^a,b^ ± 0.24	2.14 ^a^ ± 0.24	1.39 ^b^ ± 0.10
Syringic acid	0.54 ^a^ ± 0.08	0.38 ^a,b^ ± 0.04	0.23 ^b^ ± 0.02
Vanillic acid	0.18 ^a^ ± 0.02	0.22 ^a^ ± 0.02	nd
Cinnamic acid	3.98 ^a^ ± 0.26	4.46 ^a^ ± 0.36	5.08 ^a^ ± 0.47
*p*-Hydroxybenzoic acid	nd	0.24 ^a^ ± 0.04	0.08 ^b^ ± 0.02
Sum of phenolic acids	17.75	17.88	18.49

Values in the same row followed by the same letter are not significantly different on the basis of Tukey’s honestly significant difference (THSD) test (*p* < 0.05).

**Table 3:** The antioxidant activity of propolis extracts at the concentration of 0.1 mg/mL.

Propolis Extracts	DPPH· Free-Radical-Scavenging Activity (%)	Fe^3+^-Reducing Power (Ab = 700 nm)	Ferrous Ion (Fe^2+^)-Chelating Activity (%)
EPA	31.25 ^a^ ± 3.73	1.07 ^a^ ± 0.05	43.33 ^a^ ± 0.94
EEP70	29.38 ^a^ ± 4.06	0.96 ^a^ ± 0.06	43.00 ^a^ ± 1.63
EEP96	30.44 ^a^ ± 1.83	1.00 ^a^ ± 0.08	34.00 ^b^ ± 4.32
Standards	Trolox 40.22 ± 3.64	Trolox 1.60 ± 0.05	EDTA 77.33 ± 2.87
	BHT 31.67 ± 2.71	BHT 1.27 ± 0.01	

Ab = absorbance. Values in the same column followed by the same letter are not significantly different on the basis of Tukey’s honestly significant difference (THSD) test (*p* < 0.05). BHT (butylated hydroxyltoluene) and Trolox were used as the standard antioxidants; EDTA (ethylenediaminetetraacetic acid) was used as the ferrous ions standard chelator.

**Table 4:** Hemolytic activity of propolis extracts (at 0.1 mg/mL after 1 h at 37 °C) and their cytoprotective effects (at 0.05 mg/mL) against 2,2′-azobis-(2-methylpropionamidine) dihydrochloride (AAPH)-induced oxidative hemolysis.

Propolis Extracts	Hemolysis (%) */Dominated RBC Shape	Oxidative Hemolysis Protection (%)
EPA	3.16 ^a^ ± 1.44/D	76.80 ^a^ ± 7.37
EEP70	2.94 ^a^ ± 1.11/D	61.40 ^b^ ± 12.50
EEP96	3.04 ^a^ ± 1.37/D	76.80 ^a^ ± 7.58
Standards	Trolox 2.73 ± 1.50	Trolox 90.01 ± 6.69
	BHT 3.38 ± 1.86	BHT 33.00 ± 5.57

* Hemolysis values of less than 5% denote no hemolytic activity of the compound studied. D—discocytes, biconcave red blood cells (RBCs) as control cells. Values in the same column followed by the same letter are not significantly different on the basis of Tukey’s honestly significant difference (THSD) test (*p* < 0.05).

**Table 5:** The effect of flavonoid concentrations in propolis extracts on antioxidant activity.

Flavonoids	DPPH· Free-Radical-Scavenging Activity	Fe^3+^-Reducing Power	Ferrous Ion (Fe^2+^)-Chelating Activity	Oxidative Hemolysis Protection
Apigenin	0.9294	0.7719	0.0083	0.9795
Chrysin	0.5739	0.2944	0.0347	0.9370
Galangin	0.3893	0.1267	0.4649	0.7434
Kaempferol	0.5739	0.2944	0.0347	0.9370
Pinocembrin	0.5739	0.2944	0.0347	0.9370
Quercetin	0.3398	0.2422	0.1441	0.7630

**Table 6:** Minimal inhibitory concentration (mg/mL) of propolis extracts.

Fungal Strain	MIC (mg/mL)			
	EPA	EEP70	EEP96	3-Iodo-2-propynylbutylcarbamate
*Aspergillus niger*	7.5	7.5	7.5	0.75
*Aspergillus versicolor*	2.0	2.0	2.0	0.75
*Paecilomyces variotii*	2.0	5.0	7.5	1.0
*Penicillium funiculosum*	7.5	5.0	5.0	1.0
*Trichoderma virens*	5.0	5.0	5.0	1.0
*Penicillium cyclopium*	1.0	1.0	1.5	0.75
*Aureobasidium* *pullulans*	0.5	0.5	1.0	1.0

**Table 7:** The effect of flavonoid concentrations in propolis extracts on antifungal activity.

Flavonoids	Apigenin	Chrysin	Galangin	Kaempferol	Pinocembrin	Quercetin
*p*-Value of the Wilks test	0.0048	0.0000	0.0013	0.0000	0.0000	0.8934

## Supplementary Materials

The following are available online. Table S1: Multiple reaction monitoring (MRM) conditions of the polyphenols determined by UPLC/ESI/MS/MS.
