# Supplementary material for: Effect of the Solvent on Propolis Phenolic Profile and its Antifungal, Antioxidant, and In Vitro Cytoprotective Activity in Human Erythrocytes Under Oxidative Stress
Source: Molecules. 2020 Sep 17;25(18):4266. doi: 10.3390/molecules25184266 (PMC7571116; doi:10.3390/molecules25184266)
Supplement: Supplementary file 1 [file molecules-25-04266-s001.pdf]

Table S1

Multiple Reaction Monitoring (MRM) conditions of the polyphenols determined by UPLC/ESI/MS/MS

Compound	Ion mode	Parent ion (m/z)	Primary daughter ion (m/z)	Secondary daughter ion (m/z)	Collision energy (eV)
Pinocembrin	poz	257.0	213.0	153.0*	30
Galangin	neg	269.0	241.0*	227.0	29
Kaempferol	neg	284.8	117.0	93.4*	42
Apigenin	neg	269.0	225.0*	151.0	35
Quercetin	neg	300.9	179.0	151.0*	22
Rutin	neg	609.0	300.1*	271.1	43
Epicatechin	neg	289.1	245.1	109.1*	25
Catechin	neg	289.1	245.1*	109.1	23
Caffeic acid	neg	179.0	135.1*	89.1	19
Coumaric acid	neg	163.0	119.0*	93.1	21
Vanillic acid	poz	166.8	137.0*	109.1	16
Ferulic acid	poz	195.1	176.9	132.0*	19
Chrysin	neg	252.9	225.0	208.9*	40
Naringenin	neg	271.1	151.1*	119.2	25
Pinobanksin	neg	271.0	253.0*	225.0	27
Genistein	neg	269.0	195.0	133.0*	32
Pinostrobin	poz	271.1	225.1*	167.0	34
Myricetin	neg	316.8	151.1*	137.1	27
Cinnamic acid	neg	146.8	102.6*	77.0	15
Chlorogenic acid	neg	353.1	191.1*	85.1	25
p-hydroxybenzoic acid	neg	137.1	93.2*	65.2	18
Sinapic acid	poz	223.0	192.9	163.9*	26
Syringic acid	poz	199.0	155.1	140.0	20

*Transitions used for quantification
